# Visualization of spatial inhomogeneity in the superconducting gap using micro-ARPES

**DOI:** 10.1080/14686996.2024.2379238

**Published:** 2024-08-05

**Authors:** Yudai Miyai, Shigeyuki Ishida, Kenichi Ozawa, Yoshiyuki Yoshida, Hiroshi Eisaki, Kenya Shimada, Hideaki Iwasawa

**Affiliations:** a Graduate School of Advanced Science and Engineering, Hiroshima University, Higashi-Hiroshima, Japan; b Research Institute for Advanced Electronics and Photonics, National Institute of Advanced Industrial Science and Technology, Ibaraki, Japan; c Institute of Materials Structure Science, High Energy Accelerator Research Organization (KEK), Tsukuba, Ibaraki, Japan; d Graduate Institute for Advanced Studies, SOKENDAI, Tsukuba, Ibaraki, Japan; e Research Institute for Synchrotron Radiation Science (HiSOR), Hiroshima University, Higashi-Hiroshima, Japan; f International Institute for Sustainability with Knotted Chiral Meta Matter (WPI-SKCM^2^), Hiroshima University, Higashi-Hiroshima, Japan; g Research Institute for Semiconductor Engineering (RISE), Hiroshima University, Higashi-Hiroshima, Japan; h Synchrotron Radiation Research Center, National Institutes for Quantum Science and Technology (QST), Sayo, Japan; i NanoTerasu Center, National Institutes for Quantum Science and Technology (QST), Sendai, Japan

**Keywords:** Angle-resolved photoemission spectroscopy (ARPES), spatially-resolved ARPES, micro-ARPES, gap inhomogeneity, high-Tc cuprates, measurement informatics

## Abstract

Electronic inhomogeneity arises ubiquitously as a consequence of adjacent and/or competing multiple phases or orders in strongly correlated electron systems. Gap inhomogeneity in high-Tc cuprate superconductors has been widely observed using scanning tunneling microscopy/spectroscopy. However, it has yet to be evaluated by angle-resolved photoemission spectroscopy (ARPES) due to the difficulty in achieving both high energy and spatial resolutions. Here, we employ high-resolution spatially-resolved ARPES with a micrometric beam (micro-ARPES) to reveal the spatial dependence of the antinodal electronic states in optimally-doped Bi 2Sr 2CaCu 2O 8+δ. Detailed spectral lineshape analysis was extended to the spatial mapping dataset, enabling the identification of the spatial inhomogeneity of the superconducting gap and single-particle scattering rate at the micro-scale. Moreover, these physical parameters and their correlations were statistically evaluated. Our results suggest that high-resolution spatially-resolved ARPES holds promise for facilitating a data-driven approach to unraveling complexity and uncovering key parameters for the formulation of various physical properties of materials.

## IMPACT STATEMENT

This work pioneers high-resolution micro-ARPES for the first-time quantitative mapping and analysis of micro-scale superconducting gap inhomogeneity in high-*T_c_* cuprate superconductors.

## GRAPHICAL ABSTRACT

**Figure:**
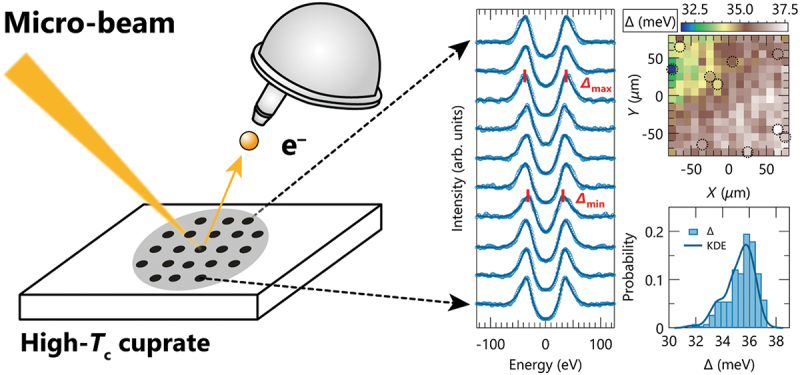

## 1. Introduction

The emergence of quantum phenomena often coincides with dominant states that lack spatial homogeneity, originating from the cooperative or competing interplay among internal degrees of freedom such as charge, spin, orbital, and lattice [1]. This complexity often results in the formation of local structures with various sizes and scales through a process known as self-organization, which is observed ubiquitously in strongly correlated materials. Understanding the physical properties of such quantum materials requires unraveling the interconnection between spatial and electronic inhomogeneity.

One representative example is high-Tc cuprate superconductors, which exhibit gap inhomogeneity extensively studied using scanning tunneling microscopy/spectroscopy (STM/STS) [2–5]. These studies have revealed nano-scale gap inhomogeneity, shedding light on the intricate electronic structures of cuprates. However, in terms of the momentum (k) dependent behavior expected due to the d-wave superconducting symmetry in cuprates, the k-integrated information is not adequate to address how gap inhomogeneity appears in real and k-spaces. Instead, angle-resolved photoemission spectroscopy (ARPES) [6,7], which offers insights into the k-resolved gap properties in contrast to tunneling techniques, though the spatial resolution of ARPES has been poor until recently and thus insufficient to study local electronic structures. In recent years, however, spatially-resolved APRES, often referred to as micro-ARPES or nano-ARPES has been well and increasingly developed [8–10]. However, spatially-resolved APRES has also faced challenges in studying real-space gap behavior due to the difficulty in realizing spatial resolution alongside high energy and momentum resolutions.

Here, we address this limitation by employing high-resolution micro-ARPES to identify the spatial inhomogeneity of the superconducting gap in optimally-doped Bi2212. By extending detailed spectral lineshape analysis to spatial mapping datasets, we quantify and map the superconducting gap and single-particle scattering rate. Our findings highlight the advantage of high-resolution micro-ARPES in revealing micro-scale gap inhomogeneity and enabling the statistical evaluation of physical parameters and their correlations.

## 2. Experiment

We performed high-resolution scanning micro-ARPES experiments on optimally-doped Bi-based high-Tc cuprate Bi 2Sr 2CaCu 2O 8+δ (Bi2212). High-quality optimally-doped Bi2212 (Tc = 92 K) single crystals were prepared by the traveling-solvent floating-zone technique [11]. The scanning micro-ARPES experiments were conducted at BL-28A of Photon Factory, KEK, using a high-resolution hemispherical electron analyzer (DA30, Scienta Omicron) equipped with a deflector function enabling two-dimensional angle mapping without mechanical rotation of the sample. All the data were measured using circular polarized light of 50 eV at 6.7 K, with a beam spot size of ϕ∼10 μm focused by Kirkpatrick-Baez mirror optics [12], after cleaving the samples insitu in an ultrahigh vacuum better than 8 × 10 −11 Torr at 7.5 K. We employed the circular polarization to avoid the significant suppression of spectral weight along the high symmetry lines that occurs with linear polarizations (vertical/horizontal) [13]. In addition, the circular polarization provides higher intensity compared to the linear polarizations at this beamline, which is a critical factor for achieving high-resolution spatial mapping experiments. The energy and angular resolution were set to better than 15 meV and 0.1 ∘, respectively, which are sufficiently high to determine the superconducting gap on an energy scale of a few tens of meV. It should be noted that we assessed the energy drift of the incident light to evaluate the intrinsic spatial dependence of the superconducting gap through two procedures: (1) determining the energy shift of the band edge along the nodal direction from repeated Fermi surface mappings, and (2) evaluating the energy shift of the mean value of the superconducting gap from repeated spatial mappings. The energy drift was determined to be 0.4∼0.5 meV per hour, resulting in a shift of approximately 3 meV during the spatial mapping experiments. This shift is roughly half the magnitude of the observed fluctuations in the superconducting gap, as demonstrated later. Hence, we did not include energy drift calibration in the present analysis, although it’s worth noting that the intrinsic magnitude of gap fluctuations may be half of the present results.

## 3. Results and discussions

**Figure 1.**
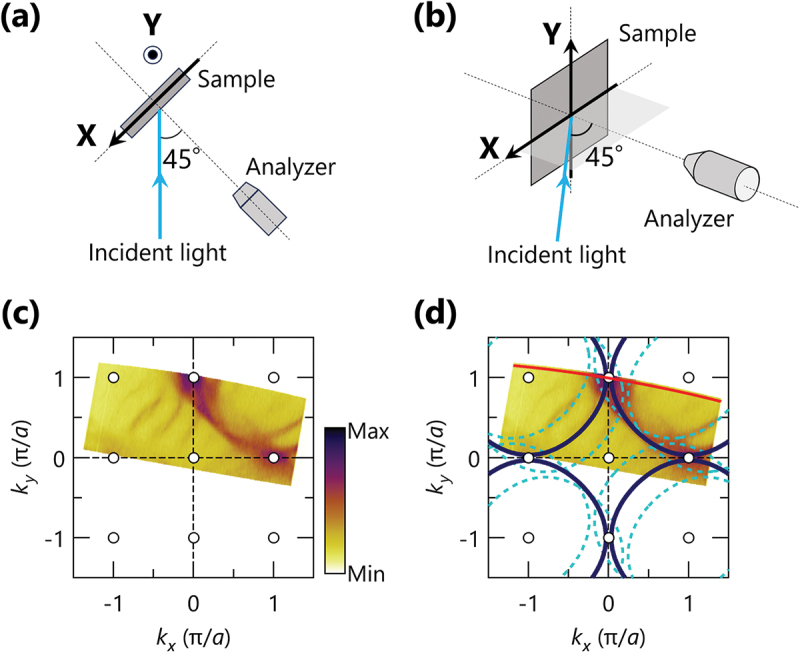
(a) and (b) Top and aerial views of the experimental configuration of the present high-resolution scanning micro-ARPES experiments, respectively. (c) Fermi surface map of Bi2212, taken at a photon energy of 50 eV with circular polarization, where the ARPES intensity was integrated within ±50 meV relative to the fermi level (EF). (d) Schematic drawing of the fermi surface of Bi2212, where the solid and dashed lines indicate the main CuO 2 band and their first diffraction replicas, respectively. For simplicity, the bilayer splitting of the CuO 2 band as well as the higher-order (2, 3…) diffraction replicas are omitted. The red line indicates the momentum location used for the spatial gap mapping at the antinodal region.

**Figure 2.**
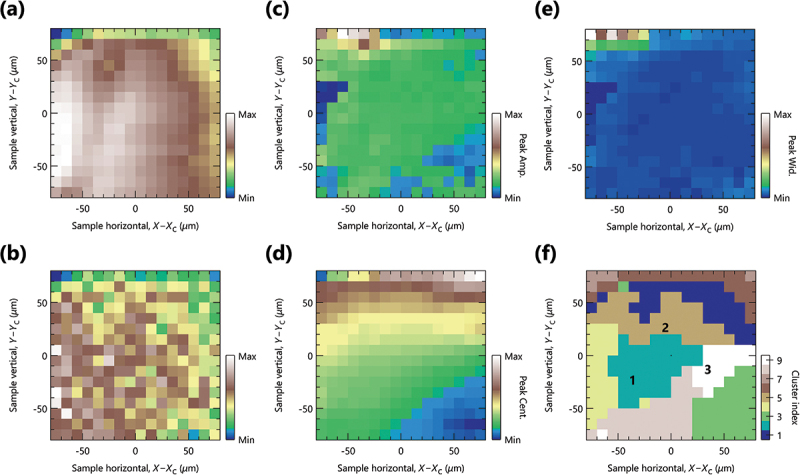
(a) and (b) scanning photoemission microscope (SPEM) image, obtained by integrating the ARPES intensity with full and limited energy and angluar windows, respectively. The integration window used in (b) is indicated by while solid lines in fig. 3(a1)-(a3). The SPEM image was measured on the 150×150 μm 2 sample surface with 10 μm steps in both the sample’s horizontal (X) and vertical (Y) directions, using a photon energy of 50 eV with circular polarization near the antinodal region in momentum space. (c)-(e) spatial distribution of the peak amplitude, center, and width, respectively, obtained by fitting angular distribution curves (ADCs) at EF±50 meV with a Lorentzian function at each position. (f) Spatial distribution of cluster indices obtained by K-means clustering using the ADCs with the maximum number of clusters (nk=9).

Figure 1(a) and (b) illustrate the experimental setup of the present micro-ARPES experiment, based on scanning photoemission microscopy (SPEM) [8–10]. In SPEM experiments, an ARPES image, I(Ek, θ), is sequentially measured at spatial coordinates while mechanically scanning the sample surface. Here, Ek and θ are the kinetic energy (Ek) and emission angle (θ), respectively. In this study, we performed the spatial ARPES mapping in two spatial dimensions, xi and yj, along the horizontal and vertical axes of the sample (X and Y), resulting in the four-dimensional dataset I(Ek, θ, xi, yj). Here, i (j) is an integer ranging from 1 to nx (ny), representing the number of acquisition points along the X (Y) axis. The step sizes of both X and Y axes were set to 10 μm, comparable to the spatial resolution of the present micro-ARPES system [12]. The spatial distribution of ARPES intensity is often visualized as a so-called SPEM image, obtained by integrating each ARPES image within specific energy and angular windows (see Figure 2(a)). It should also be noted that the spatial ARPES mapping is performed essentially at a specific and fixed momentum space.^1^ As our focus is to clarify the inhomogeneity in the superconducting gap, we set the region of interest of this work to the antinodal region (π, 0), as the d-wave superconducting gap evolves from zero to maximum along the nodal region (0,0)-(π,π) towards the antinodal region. The momentum location should thus be determined prior to conducting the spatial mapping in the scanning micro-ARPES experiments, which is typically achieved by measuring the Fermi surface.

**Figure 3.**
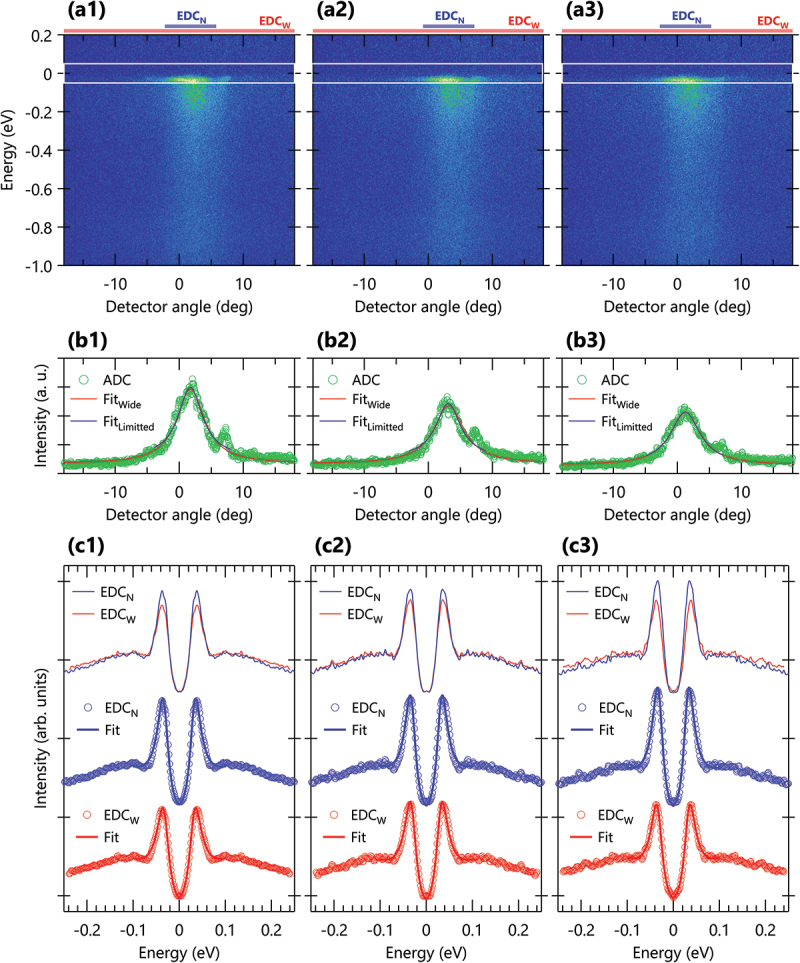
(a1)-(a3) the ARPES intensity plots at several sample positions on the cleaved surface. (b1)-(b3) the angular distribution curves (ADCs) obtained by integrating ±50 meV with respect to EF, along with the fitting results using Lorentzian functions with the wide (red) and limited (blue) fitting range. The integration ranges are indicated by the white boxes in fig. 3(a1)-(a3). (c1)-(c3) symmetrized energy distribution curves (EDCs), where the spectra are normalized by the area within ±0.2 eV with respect to EF after subtracting the constant background at EF. Symmetrized EDCs are fit by the phenomenological spectral function, as described in the text. The EDCs, labeled as EDC N and EDC W, are obtained by integrating the narrow and wide angular windows (±4 ∘ and ±18 ∘), respectively, relative to the main peak position of ADCs in fig. 3(b1)-(b3). The narrow and wide integration areas are indicated by blue and red horizontal bars, respectively, at the top of Fig.3(a1)-(a3). Note that the numbers 1–3 in the figure labels correspond to sample positions on the cleaved surface, as respectively indicated by numbers (1, 2, and 3) in fig. 2(f).

Figure 1(c) displays the Fermi surface (FS) of Bi2212 taken at a photon energy of 50 eV with circular polarization, where the ARPES intensity is integrated within EF±50 meV. The observed FS sheets predominantly consist of the single CuO 2 main band as well as their first-order diffraction replicas, while neither bilayer splitting nor higher-order (2, 3, …) diffraction replicas are obviously observed. These features are essentially captured by the schematic FS drawn in Figure 1(d), where the red line indicates the momentum cut crossing the antinodal region, employed for the spatial mapping.^2^ Note that we treat the CuO 2 band as the single band in the subsequent analysis of the superconducting gap. Here, the spectral weights near the antinodal region are dominated by the antibonding band rather than the bonding band, as a consequence of the significant dependence of spectral weights due to matrix element effects, involving photon energy, polarization, and momentum space [14]. On the other hand, it should be emphasized that the presence of first-order diffraction replicas requires careful consideration. Whereas the main and replica bands exist closely near the antinodal region, they exhibit different gap properties due to the translation of replica bands from the original momentum space by the incommensurate modulation vector Q = (0.21π, 0.21π) [15,16]. We will discuss this issue later in the subsequent analyses in Figures 2 and 3.

Having identified the target antinodal location experimentally (red line in Figure 1(c)), we investigate the spatial modulations of electronic states of Bi2212 along there by high-resolution scanning micro-ARPES. The spatial ARPES mapping was performed on a 150×150 μm 2 local area with 10 μm steps in both the sample’s horizontal (X) and vertical (Y) directions, using a photon energy of 50 eV with circular polarization. Note that the scanning was unidirectionally performed along the X axis while stepping on the Y axis, to suppress uncertainty due to the backlash of the motors and enhance the reproducibility of sample position [17].

We visualize the spatial distribution of the antinodal electronic states by utilizing the SPEM images, as shown in Figure 2(a) and (b), obtained by integrating over the whole and limited energy and angular windows, respectively. The pixel intensity represents the integrated spectral weight of ARPES spectrum, as exemplary shown in Figure 3(a1)-(a3), where the white rectangles indicate the limited integration windows used for Figure 2(b). While the limited integration windows make the SPEM image more sensitive to the near-EF electronic states, we found essentially similar spatial appearances in Figure 2(a) and (b). This indicates that the antinodal electronic states were observed rather homogeneously in this local region.

To extract more detailed spectral information, we analyzed each ARPES spectrum using the angular distribution curves (ADCs) within the energy of EF±50 meV at each spatial coordinate. The representative ARPES spectra and ADCs are shown in Figure 3(a1–3) (b1–b3), respectively, where the spectral information of ADCs was extracted by fitting a Lorentzian function.^3^ Two types of fitting were performed with the wide (±18 ∘) and narrow (±4 ∘) fitting range relative to the main peak position (red and blue lines), as shown in Figure 3(b1–3). Since the fitting errors of peak position were smaller when using the wider fitting range, we employed the wider fitting range for the subsequent analysis. The fitting results are summarized as the spatial maps of peak amplitude, center, and width, as respectively shown in Figure 2(c–e).

Along the antinodal direction, the intense and flat bands are observed in the vicinity of EF, accompanied by two sub-bands on the left and right of the main peak, as shown in Figure 3(a1–3) and 3(b1)-(b3). The intense peak is effectively a merge of the two main CuO 2 bands, while the sub-bands consist of the first-order diffraction replicas. Note that the left sub-band is not visible, possibly due to matrix-element effects. At first sight, peak shifts along the angular direction are apparent depending on the sample position. However, the observed shifts (Figure 2(d)) imply a misalignment between the manipulator and detector’s focal axes because the peak center, corresponding to the high-symmetry ΓM line, should not be varied against the sample coordinates (xi and yj). Nevertheless, the possible misalignment effects would give insignificant effects on the antinodal electronic states, because peak amplitudes and widths as seen in Figure 2(c,e) exhibit more uniform spatial distributions across most of the spatial area. It is also worth noting that the present spatial mapping dataset can be grouped as shown in Figure 2(f), by the K-means method using the ADCs [18] with the maximum number of clusters (nk=9).^4^ Whereas the clustering facilitates the identification of the spatial evolution, the spatial distribution of the clustering index primarily reflects peak positions, and may not fully reflect intrinsic electronic properties in this case. On the other hand, such peak shifts induce complications on the spatial gap analysis, as the extraction of energy distribution curves (EDCs) becomes less straightforward and requires adjustment of the integration angular window at each position for proper evaluation.

To take into account the observed peak shifts, we performed a step-by-step spectral analysis by combining the analysis of ADCs and EDCs. First, we extracted the EDCs with two different angular windows relative to the peak center of the ADCs, as determined in Figure 3(b1)-(b3). The narrow and wide integration windows (±4 ∘ and ±18 ∘) are indicated by the blue and red horizontal bars, respectively, in the upper part of Figure 3(a1)-(a3). Next, we symmetrized the extracted EDCs, to remove the thermal broadening effect [19]. For comparison, the EDCs were normalized by the area between ±0.2 eV after subtracting the constant backgrounds at EF±5 meV. The resulting EDCs, shown in Figure 3(c1)-(c3), are labelled by the angular integration windows, as EDC N and EDC W.

It is obvious that there are no significant differences in the overall spectral features between EDC N and EDC W, as observed in Figure 3(c1)-(c3). To quantify the superconducting gap, we fit the EDCs with a widely-used phenomenological form [20],

(1) $$I\mathrm{DOS}(\omega )\propto \frac{\Sigma \prime \;\prime (kF,\omega )}{\left[\omega -\Sigma \prime (kF,\omega )\right]2+\Sigma \prime \;\prime \left(kF,\omega \right)2}$$

using the model self-energy Σ(kF,ω)=−iΓ1+Δ2/ω, where Δ and Γ1 represents the superconducting gap size and single-particle scattering rate, respectively. The constant and linear backgrounds are also included as IBG(ω)=ICBG+ILBG(ω), where ICBG is the constant and ILBG(ω)=aω is the linear background while the sign of slope ‘a’ changes between positive and negative for ω>0 and ω<0. It should be noted that claims have recently arisen regarding Eq. 1 as a minimal model to reproduce the gap spectrum observed by ARPES [21]. It has been suggested that this equation is not adequate for high-Tc cuprates, where the self-energy should be expressed as Σ(kF,ω)=−iΓ1+Δ2/(ω+Γ0), by including the pair-breaking rate Γ0. Nevertheless, we employed Eq. 1 in this work for simplicity and regard the effects of Γ0, which fills the spectral weight around EF, as somehow included in ILBG.

As evident in Figure 3(c1)-(c3), all the EDCs (circles) are almost perfectly reproduced by the fitting curves (solid lines). Then we compare the gap size obtained by fitting EDC N and EDC W (ΔN and ΔW): negligible differences were found in Figure 3(c1) and (c2) as (ΔN, ΔW) = (35.4±0.1, 35.4±0.1) and (34.6±0.1, 34.5±0.1), while a slight variation was observed in Figure 3(c3) as (34.6±0.1, 36.2±0.1), where Δ is in a meV unit. Besides that the sharper quasiparticle peaks are apparent in EDC N compared to EDC W and that EDC N is expected to be more sensitive to the main CuO 2 band, we employed the EDC N in the following evaluation of the spatial distribution of superconducting gap.

**Figure 4.**
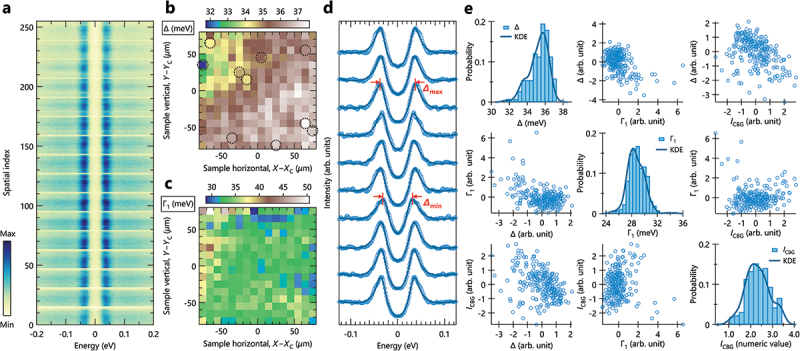
(a) Symmetrized EDC N‘s as a function of energy on the bottom axis and spatial index on the left axis. (b) and (c) spatial maps of the superconducting gap Δ and single-particle scattering rate Γ1, respectively. (d) Exemplary symmetrized EDC N‘s extracted from several points, accompanied by the fitted curves (solid lines). The points are randomly selected except to include the points giving the maximum and minimum of the size of superconducting gap. (e) Scatter-plot matrix for Δ, Γ1, and the constant background I0. Each off-diagonal plot in the matrix represents the correlation between two variables, while the diagonal plots show the distribution of each variable through histograms, along with kernel density estimation (KDE). Here, the data used for the scatter plots are standardized, while the raw data are used for the histograms.

Figure 4 summarizes the results of an extended gap analysis for all the spatial mapping data. The symmetrized EDC N’s at all coordinates (xi,yj) are displayed as an image in Figure 4(a), where 2D coordinates are simplified into a 1D spatial index n (=nx×ny), where nx=ny=16, giving n=256 in the present case. One can directly see that high-intensity parts, corresponding to quasiparticle peaks, modulates along the spatial index. This means that the gap size is also varied depending on the spatial coordinates. By fitting all the symmetrized EDC N’s, we can map out the superconducting gap Δ and single-particle scattering rate Γ1, as shown in Figure 4 (b,c), respectively. Our spatial gap analysis can thus identify the micro-scale spatial fluctuations both in Δ and Γ1. The observed gap fluctuations can be more clearly visualized in the exemplary symmetrized EDCs, as shown in Figure 4(b), where the spatial coordinates are picked up randomly, except to include the points where the gaps are at their maximum and minimum. At first glance, the image plots of Δ and Γ1 seem to exhibit opposite spatial distributions. It is, however, difficult to quantify the correlation between these two physical parameters solely from the image intensity.

To perform statistical evaluation of Δ and Γ1 and their correlation, we employed scatter-plot matrices as shown in Figure 4(e), which are useful for visualizing and quantifying the pairwise relationship between multiple variables. Note that the constant background ICBG is also plotted in addition to Δ and Γ1, as ICBG is somehow related to the pair-breaking rate Γ0 in terms of filling the gap. The diagonal plots show the distribution of these three parameters through histograms, while the off-diagonal plots are the scatter plots of two variables, illustrating the correlation between them. Here, the raw values are used for histograms, while standardized values are used for scatter plots.

We evaluated the linear relationship between variables using Pearson correlation coefficient (r), along with the evaluation of the statistical significance of r via p-value.^5^ One can see a gradual slope from the upper left to the lower right, representing the weak negative correlation between Δ and Γ1 as well as Δ and ICBG, as indicated by r(Δ,Γ1)=−0.43 (p=0.0), r(Δ,ICBG)=−0.42 (p=0.0), respectively. In contrast, no correlation was observed between Γ1 and ICBG, as seen in r(Γ1,ICBG)=0.0 (p=0.7), where the high p-value stems from the difficulty in handling circular distribution of data points. Typically, a systematic studies, such as the doping dependence on various samples, are required to gain insights on the relation among the physical parameters. Alternatively, our spatial spectral analysis enables this from one sample alone, demonstrating its great advantage in pursuing a key ingredient of high-Tc in cuprate superconductors.

Another advantage of the present analysis is that it enables the statistical evaluation of the gap parameters, such as Δ=35.2±1.1 meV and Γ1=28.7±1.3 meV, estimated from the mean value and standard deviation. By comparing the mean value and the most frequent value (or mode) seen in the histograms, what is observed is the deviation for Δ while the agreement for Γ1. This simultaneously indicates the non-negligible effects of outliers in Δ within the present dataset. In such a case, the clustering analysis, one of the representative unsupervised learning techniques, is a feasible approach to evaluate only the representative values while isolating the outliers, as we demonstrated in Ref [22]. In the present case, however, the number of data points are inadequate for statistically meaningful evaluation, as the present sample size (n=256) is almost close to the limit,^6^ to detect a significant correlation coefficient in the order of 0.2, indicating the clustering with the maximum number of clusters larger than one (nk>1) might yield meaningless results statistically. Therefore, we avoid utilizing clustering analysis to maintain the sample size for statistical evaluation. Despite such incompleteness of the analysis, our results highlight several great advantages of the present approach utilizing spatially-resolved ARPES. It not only allows us to identify spatial distributions of physical parameters but also enables us to presume the `true’ value of physical parameters by identifying the mean, median, and most frequent values as well as the standard deviation.

Finally, we discuss the origin of the observed spatial inhomogeneity of Δ at the micro-scale. In our recent laser micro-ARPES study along the nodal direction on overdoped Bi2212, the spatial inhomogeneity was also observed at similar length scales in various physical parameters, including Fermi velocity, Fermi momentum, and coupling constant [22]. Since the Fermi momentum kF is the indicator of the doping level of the system, the present results may be induced by the spatial doping inhomogeneity. Indeed, our preliminary results on the Fermi surface area measured at slightly different spatial coordinates suggest the variations in the doping level (see Appendix A). Accordingly, the observed gap inhomogeneity, at first glance, appears similar to the nano-scale gap inhomogeneity widely reported by STM/STS [2–5], where oxygen dopants are discussed as being responsible for the disorder in charge potentials, altering the local electronic environment. However, we should point out that there are critical differences between the ARPES and STM/STS observations in both qualitative and quantitative manners. From a qualitative viewpoint, spatially-resolved ARPES provides momentum-resolved information, while STM/STS is momentum-integrated. The momentum-resolved spatial ARPES mapping, which we proposed in this work, is powerful for understanding how the electronic states behave both in the real and momentum spaces. Moreover, we observed a relatively small gap inhomogeneity of about 30–40 meV, while STM/STS observed a much larger inhomogeneity of about 20–70 meV. Considering the gap magnitude, the present results should be more sensitive to the superconducting gap, while the STM/STS results may reflect contributions from both the superconducting gap and the pseudogap. We should also note that the atomic-level inhomogeneity observed by STM/STS can be regarded as homogeneous on a much larger length scale, by a few thousand times, as in the present micro-ARPES case. Hence, the spatial inhomogeneity observed by ARPES should be considered a different type of inhomogeneity compared to those observed by STM/STS, although there is a possibility that they share the same origin, such as oxygen dopant inhomogeneity. Consequently, our results demonstrate the superconducting gap inhomogeneity on the micro-scale in cuprates for the first time. In addition, the observed spatial fluctuation in the superconducting gap was found to be much smaller than that reported in STM/STS results, which also include pseudogap contributions.

On the other hand, further investigations are required to pin down the origin of the observed superconducting gap inhomogeneity and to understand the different inhomogeneous properties between the superconducting gap and pseudogap. These issues will be clarified by mapping the momentum space and doping levels using high-resolution micro-ARPES and high-resolution nano-ARPES, enabling the examination of nano-scale spatial inhomogeneity. To achieve this, it is necessary to enhance the experimental efficiency of the spatially-resolved ARPES techniques. One feasible approach is the integration of measurement informatics into spatially-resolved ARPES measurements, which we are currently working on.

## 4. Summary

We have performed the spatially-resolved study on the antinodal electoronic states of optimally-doped Bi2212 using high-resoltion micro-ARPES. Our spatial-spectral analysis identified gap inhomogeneity at the micro-scale and enabled the statistical evaluation of physical parameters and their correlations. This analysis is expected to facilitate a data-driven approach to understanding and identifying key parameters for the formulation of high-Tc superconductivity in cuprates.

## Figures and Tables

**Figure A1.**
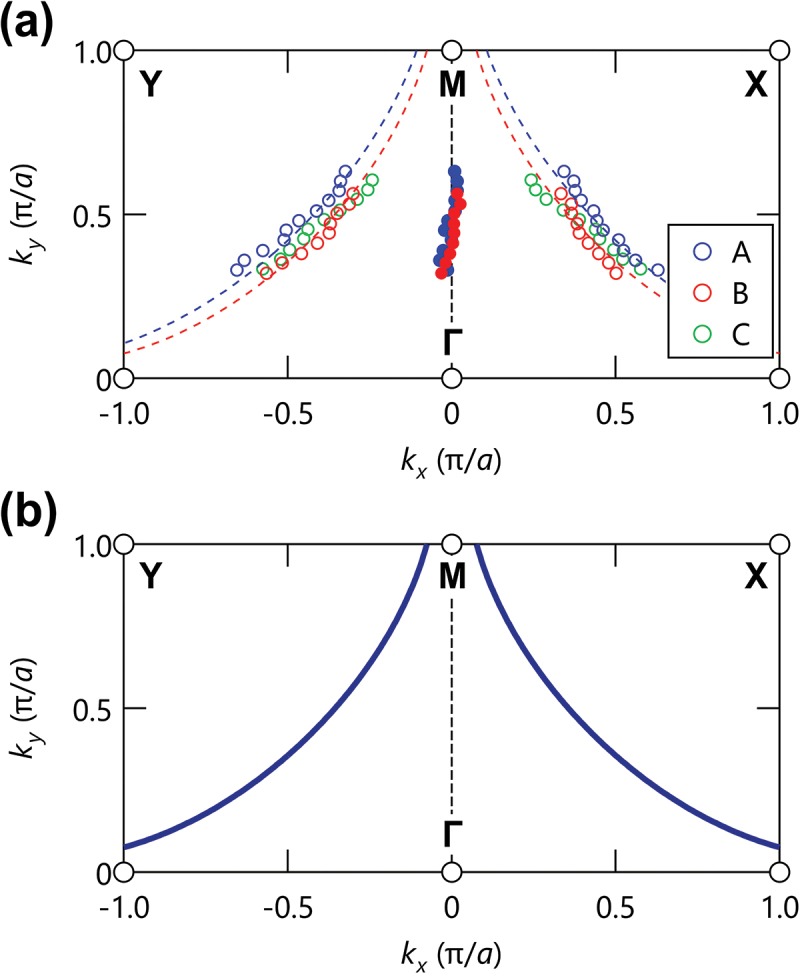
(a) The fermi momentum (kF) plots at three different sample positions, where the open circles indicate the kF positions obtained by fitting the momentum distribution curves (MDCs) at EF, while the closed circles indicate the midpoints of the positive and negative kF plots for points a and B. The dashed lines represent the guides for the eyes, obtained by magnifying (b) the schematic fermi surface of the main CuO 2 band of Bi2212.

## Notes

1. Except for potential experimental errors on emission angles caused by misalignment between the manipulator and detector axes [17].

2. Considering that the large deflection angle causes the reduction of the in-plane detection angles as well as potential image distortions, the sample was mechanically tilted to measure the antinodal direction.

3. The actual form for fitting the ADCs used was: f(θ)=a+bθ+L(θ,LA,LC,LW), where (a+bθ) is a linear background and L is the Lorentzian function with an amplitude, center, and width, represented by LA, LC, and LW, respectively.

4. The absolute determination of the maximum number of cluster nk, a hyperparameter of K-means clustering, is challenging issue. In this work, we used K-means clustering to roughly visualize the spectral evolution. Initially, we employed a large number of $n_{k}^{ini}$, and then compared the averaged ADCs within each cluster. Subsequently, we counted the number of clusters neq exhibiting almost equivalent averaged ADCs, and finally obtained nk estimated by $nk=n_{k}^{ini}-n_{k}^{eq}$.

5. The Pearson correlation coefficient r ranges from −1 to + 1, where + 1 indicates a perfectly positive correlation (as one variable increases, the other increases), while −1 indicates vice versa, and 0 indicates no correlation between the two variables. The p-value indicates the probability of obtaining the observed r. In general, a p-value less than 0.05 is generally considered statistically significant, meaning that there is less than a 5% chance that the observed correlation occurred by chance.

6. By following one common approach based on Fisher’s r–to–z transformation, the sample size required to detect a statistically significant correlation coefficient can be calculated [23].

## Appendix A. Spatial variations in doping level

In our previous work on overdoped Bi2212 (Tc = 75 K) using high-resolution laser-micro ARPES [22], we observed spatial inhomogeneities in the nodal electronic states, such as the effective mass, Fermi velocity, and coupling parameter, accompanied by variations in the nodal Fermi momentum, which can serve as an indicator of the doping level. As preliminary results on optimally-doped Bi2212 in this work, we evaluated the Fermi momentum (kF) plots at three slightly different sample positions, labeled as A, B and C. Note that the spatial differences among the three positions ranges from 20 to 70 μm in X and/or Y coordinates.

Figure 5 shows the obtained kF plots (circles) at three different positions, and the midpoints of the positive and negative kF plots (closed circles) for points A and B. Note that the kF plots are obtained by fitting the momentum distribution curves (MDCs) at EF for points A and B. For point C, the positive side of kF is obtained by symmetrizing the negative side of kF due to the difficulty in determining the peak position owing to weak intensity. As the midpoints are almost aligned to the high symmetry (0,0)-(0,π) ΓM line, the absolute value of the observed kF plots are validated. As evident in Figure A1(a), a clear spatial dependence is observed, as indicated by the guided schematic Fermi surfaces (dashed lines), which are obtained by magnifying the schematic Fermi surface of the main CuO 2 band of Bi2212 (Figure A1(b)) at different magnifications. These results suggest that the observed gap inhomogeneities are related to the doping variations on the cleaved surface.
